# Distinct emphysema subtypes defined by quantitative CT analysis are associated with specific pulmonary matrix metalloproteinases

**DOI:** 10.1186/s12931-016-0402-z

**Published:** 2016-07-26

**Authors:** Kristoffer Ostridge, Nicholas Williams, Viktoriya Kim, Stephen Harden, Simon Bourne, Ngaire A. Coombs, Paul T. Elkington, Raul San Jose Estepar, George Washko, Karl J. Staples, Tom M. A. Wilkinson

**Affiliations:** 1 Southampton NIHR Respiratory Biomedical Research Unit, Southampton General Hospital, Tremona Road, Southampton, SO16 6YD UK; 2 Department of Radiology, University Hospital Southampton, Southampton General Hospital, Tremona Road, Southampton, SO16 6YD UK; 3 Primary Care & Population Sciences, University of Southampton Faculty of Medicine, Southampton General Hospital, Tremona Road, Southampton, SO16 6YD UK; 4 Clinical and Experimental Sciences, University of Southampton Faculty of Medicine, Sir Henry Wellcome Laboratories, Southampton General Hospital, Mailpoint 810, Tremona Road, Southampton, SO16 6YD UK; 5 Department of Radiology, Laboratory of Mathematics in Imaging, Brigham and Women’s Hospital, Boston, MA USA; 6 Division of Pulmonary and Critical Care Medicine, Department of Medicine, Brigham and Women’s Hospital, Boston, MA USA; 7 Wessex Investigational Sciences Hub, University of Southampton Faculty of Medicine, Southampton General Hospital, Tremona Road, Southampton, SO16 6YD UK

**Keywords:** COPD, Emphysema, CT, Imaging, MMPs

## Abstract

**Background:**

Emphysema is characterised by distinct pathological sub-types, but little is known about the divergent underlying aetiology. Matrix-metalloproteinases (MMPs) are proteolytic enzymes that can degrade the extracellular matrix and have been identified as potentially important in the development of emphysema. However, the relationship between MMPs and emphysema sub-type is unknown. We investigated the role of MMPs and their inhibitors in the development of emphysema sub-types by quantifying levels and determining relationships with these sub-types in mild-moderate COPD patients and ex/current smokers with preserved lung function.

**Methods:**

Twenty-four mild-moderate COPD and 8 ex/current smokers with preserved lung function underwent high resolution CT and distinct emphysema sub-types were quantified using novel local histogram-based assessment of lung density. We analysed levels of MMPs and tissue inhibitors of MMPs (TIMPs) in bronchoalveolar lavage (BAL) and assessed their relationship with these emphysema sub-types.

**Results:**

The most prevalent emphysema subtypes in COPD subjects were mild and moderate centrilobular (CLE) emphysema, while only small amounts of severe centrilobular emphysema, paraseptal emphysema (PSE) and panlobular emphysema (PLE) were present. MMP-3, and -10 associated with all emphysema sub-types other than mild CLE, while MMP-7 and -8 had associations with moderate and severe CLE and PSE. MMP-9 also had associations with moderate CLE and paraseptal emphysema. Mild CLE occurred in substantial quantities irrespective of whether airflow obstruction was present and did not show any associations with MMPs.

**Conclusion:**

Multiple MMPs are directly associated with emphysema sub-types identified by CT imaging, apart from mild CLE. This suggests that MMPs play a significant role in the tissue destruction seen in the more severe sub-types of emphysema, whereas early emphysematous change may be driven by a different mechanism.

**Trial registration:**

Trial registration number NCT01701869.

**Electronic supplementary material:**

The online version of this article (doi:10.1186/s12931-016-0402-z) contains supplementary material, which is available to authorized users.

## Background

Emphysema is a key feature of chronic obstructive pulmonary disease (COPD) and contributes directly to airflow obstruction. It is defined as an abnormal permanent enlargement of air spaces distal to the terminal bronchioles, accompanied by destruction of alveolar walls [1]. Three distinct pathological sub-types of emphysema are classified according to the distribution around the secondary pulmonary lobule and are termed centrilobular (CLE), panlobular (PLE) and paraseptal (PSE) emphysema [1]. All sub-types are found in COPD patients [2, 3] although how they are distributed in individuals and populations and hence contribute to the disease is uncertain. CLE is the commonest form [3, 4] and is associated with older age [4], smoking history [4, 5] and lower FEV1 [3, 5]. PLE is common in younger age [4] and is associated with lower BMI [3, 5] and more severe GOLD stage [3]. PSE is the least common form and is associated with male sex [3], older age, worse respiratory symptoms and interstitial abnormalities [6].

Efforts have been made to study emphysema in more objective detail using CT image analysis and threshold-based quantification methods, such as %LAA_<-950_ are the current standard [7]. These techniques do not provide any information about emphysema sub-types, whereas local histogram-based emphysema (LHE) quantification is a novel method, which uses regional histogram data to quantify different emphysema sub-types on CT images [2]. This analysis has shown stronger associations with physiological and functional measures of disease than %LAA_<-950_ [2]. However, limited work has assessed the relationship between emphysema sub-types and underlying mechanisms of disease and consequently our understanding of the aetiology of these sub-types is poor. PLE in the context of alpha-1-antitrypsin deficiency (A1ATD), is perhaps the most well understood as it is the predominant sub-type in this condition where it is encoded by the Serpina 1 gene, resulting in unopposed neutrophil elastase activity [8]. In subjects without A1ATD, PLE has been linked with polymorphisms of the Serpina 2 gene [9], whilst CLE has been associated with matrix metalloproteinase-9 (MMP-9) and Transforming growth factor-β (TGF- β) polymorphisms [10] and PSE with tissue inhibitor of MMP-2 (TIMP-2) and tumour necrosis factor (TNF) polymorphisms [10].

There is growing evidence that proteases such as matrix metalloproteinases (MMPs) are important in the aetiology of emphysema. Animal models suggest a role for MMP-1 [11], -9 and -12 [12] in emphysema development while human studies demonstrate increased expression of MMP-1 [13–15], - 2 [15], -3 [15], -8 [14–16], -9 [13–19], -10 [15] and -12 [13, 20, 21] in the airways of COPD subjects. We have previously reported MMP-3, -7 and -10 were associated with overall quantitative measures of emphysema [15] while Chaudhuri found MMP-9 and -12 were associated with this measure [17, 20]. We have also previously shown that MMP-3, -7, -8, -9, -10 and -12 were associated with CT markers of small airways disease, but MMPs were not associated with bronchial wall thickening of the larger airways [15]. Despite the evidence linking MMPs to COPD globally, the specific role of MMPs in the different emphysema sub-types have not been investigated. A further complicating factor in understanding the role of MMPs in emphysema is the role of proteinase inhibitors. MMPs are tightly regulated by endogenous inhibitors, the four Tissue inhibitors of MMPs (TIMPs) [22]. Sputum MMP-9/TIMP-1 ratio has been found to be significantly raised in COPD [19], although other studies do not show increased airway ratios of MMPs/TIMPs [13, 20, 23].

In this study we used novel CT analysis of LHE patterns to systematically characterise emphysema sub-types in COPD subjects and ex/current smokers with preserved lung function. We combined this analysis with multiplex profiling of MMPs and TIMPs in bronchoalveolar lavage (BAL) fluid to further understand the complex relationship between these enzymes and inhibitors in the initiation and development of emphysema. Understanding specific mechanisms driving emphysema is a key step in the stratification of fundamental COPD pathology and provides a route into developing new therapies targeting particular disease endotypes.

## Methods

### Subjects

Subjects gave written informed consent and the study (ClinicalTrials.gov:NCT01701869) was approved by the South Central-Southampton B NRES Committee (12/SC/0304).

The methods have been described in detail previously [15]. Twenty-four subjects with mild-moderate COPD as defined by GOLD guidelines [24] were recruited. Post-bronchodilator spirometry was used to assess airflow obstruction with a FEV1/FVC ratio of <0.7 and an FEV1 of ≥50 % predicted value required. Eight current or ex-smokers with preserved lung function were also recruited. Subjects had at least a 10 pack year smoking history and exclusion criteria included a history of other pulmonary disease, long-term antibiotics/steroids or an exacerbation within the month prior to recruitment.

### CT scanning and quantitative image analysis

Subjects underwent volumetric CT scans of the chest in full inspiration using a Siemens Sensation 64 scanner. The imaging protocol consisted of; slice thickness 0.75 mm, slice separation 0.5 mm, tube voltage 120KV, effective mAs 90mAs (using dose modulation), collimation 0.6 mm and a pitch of 1. Images reconstructed with the B30 kernel were used for image analysis by custom software to characterise the emphysema pattern using local histogram information as previously described [2]. Briefly, regions of interest of size 24.18 × 24.18 mm^2^ were detected and automatically classified into one of six categories; non-emphysematous (NE), centrilobular emphysema by increasing severity (mild CLE, moderate CLE and severe CLE), panlobular emphysema (PLE) and paraseptal emphysema (PSE). This was applied to each CT scan, generating six continuous measures representing the lung volume percentage that was classified into each of the six patterns.

### Bronchoscopy

In each subject two lobes were targeted at bronchoscopy. These lobes were determined prior to the procedure by a thoracic radiologist reporting the lobes most and least affected by disease. BAL was performed by instilling 100 ml of 0.9 % saline into each lobe and recovered by aspiration. BAL fluid was filtered and centrifuged and the resultant supernatant was stored at −80 °C prior to analysis.

### MMP and TIMP analysis

MMP and TIMP concentrations in BAL were quantified using a microparticle based multiplex immunoassay (R&D systems, Abingdon, UK) and analysed on the Luminex 200 platform (Biorad Bioplex 200, Hemel Hempstead, UK). We analysed MMP-3, -7, -8, -9, -10 and -12 as we had previously linked these to CT parameters of disease [15] as well as TIMP 1–4 (Additional file 1).

### Statistical analysis

Analyses were performed using SPSS version 22. Mann-Whitney U and Fishers Exact tests compared data between groups. Associations between MMPs, spirometry and CT parameters were analysed using Spearman’s Correlation. Each subject had two lobes sampled and the mean concentrations between the lobes were used. For the purpose of statistical analysis, values that were below the lower limit of detection were given the value of half the concentration of detection. A *p*-value of <0.05 was considered statistically significant.

## Results

Groups were well matched for age, gender and smoking status (Table 1).

**Table 1:** Characteristics of participants included in the study

	COPD (*n* = 24)	Preserved lung function (*n* = 8)	*P* value
Age	66.0 (12.0)	56.0 (18.0)	0.064
Male	16	6	>0.999
Current smoker	11	5	0.685
Pack Years	39 (39.8)	32.25 (23.8)	0.268
FEV1 % predicted	69.00 (21.0)	108.00 (20.3)	<0.001*
FEV1/FVC	54.50 (10.8)	78.00 (9.5)	<0.001*
Mild CLE	33.8 (8.3)	31.9 (9.5)	0.44
Moderate CLE	21.3 (15.3)	7.1 (9.2)	0.008*
Severe CLE	0.9 (2.0)	0.0 (0.0)	0.008*
Panlobular	2.7 (1.1)	0.0 (0.0)	0.048*
Paraseptal	6.9 (3.9)	3.7 (3.0)	0.014*
Non-emphysema	34.6 (20.6)	53.5 (21.4)	0.011*

Values are given as medians (IQR). Tissue sub-types given as median % of CT lung volume classified as particular sub-type

Male and current smokers given as number of subjects

Tissue sub-types given for 31 subjects

*p < 0.05. Fisher’s exact test for male and current smoker. Mann-Whitney *U*-test for all other variables

### Emphysema sub-types

**Fig. 1:**
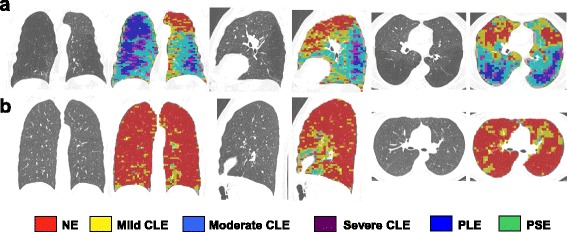
Local histogram emphysema (LHE) classification results for a coronal, sagittal and axial slice corresponding to (**a**) COPD subject and (**b**) subject with preserved lung function

CT analysis defining specific emphysema sub-types was successfully achieved in 31 subjects. Figure 1 shows a typical LHE reconstruction of a subject with COPD and preserved lung function. One scan from a subject with preserved lung function could not be analysed for technical reasons.

All subjects (COPD and preserved lung function) had non-emphysematous tissue, mild CLE, moderate CLE and PSE. Only eight subjects had >1 % of PLB. 10 subjects had >1 % of severe CLE. Non-emphysematous tissue was the most common in 15 subjects. Mild CLE was the most common in 12 subjects and moderate CLE the most common in 4 subjects.

The median percentage of each LHE pattern is shown in Table 1. Moderate CLE, severe CLE, PLE and PSE were all significantly raised in COPD subjects compared to those with preserved lung function. Non-emphysematous tissue was significantly higher in those with preserved lung function. Median percentage for mild CLE was over 30 % for subjects with and without airflow obstruction with no significant difference between the two.

There were significant associations between FEV1 % predicted and moderate CLE (rho −0.39, p0.032) and severe CLE (rho −0.37, p0.044). There were no associations between FEV1 % predicted and mild CLE (rho 0.07, p0.695), panlobular (rho −0.30, p0.097), paraseptal ( −0.31, p0.094) or NE (rho 0.32, p0.075).

### MMP and TIMP concentrations

**Fig. 2:**
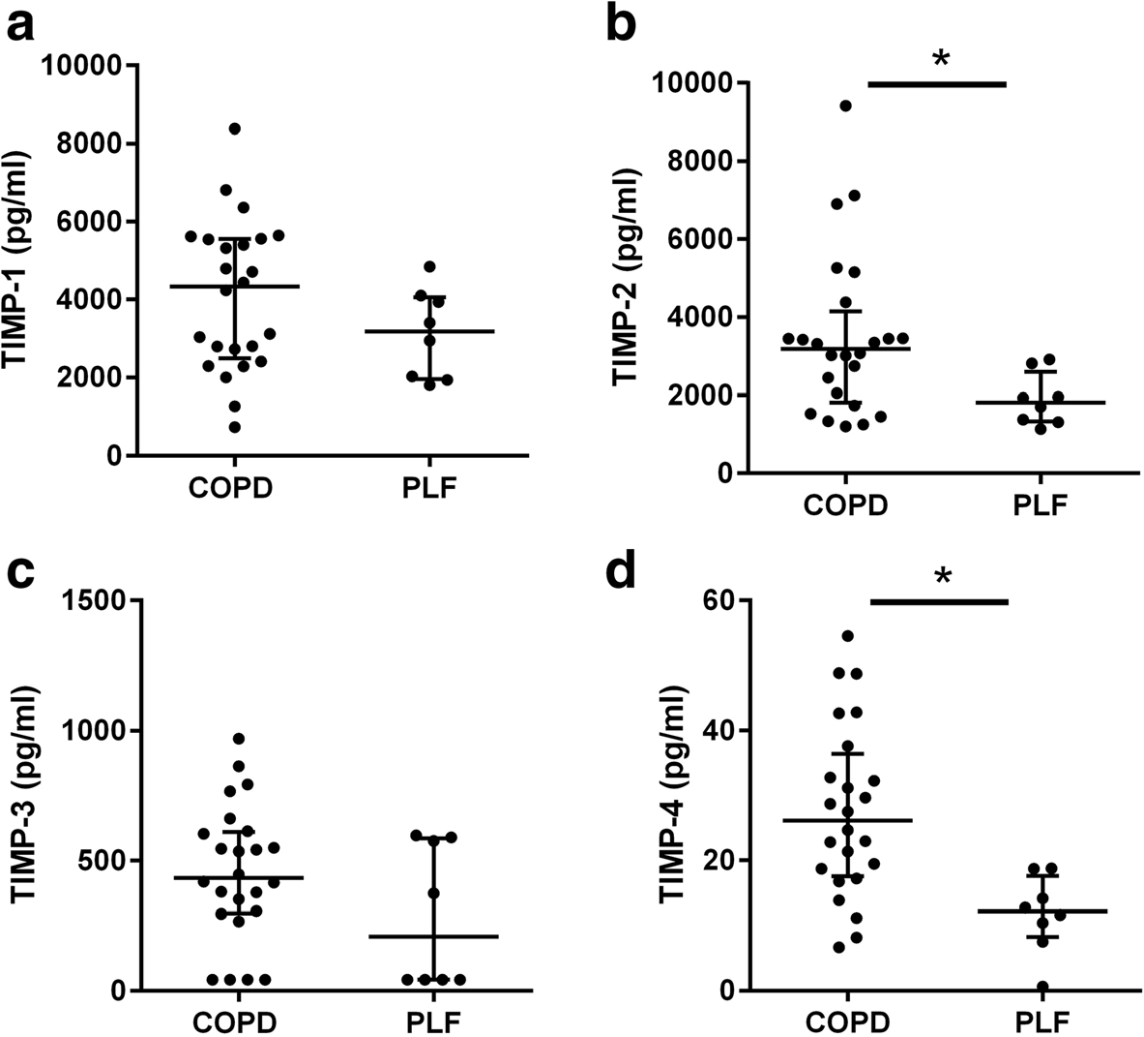
BAL expression of TIMPs in subjects with COPD and preserved lung function. **a** TIMP-1 (**b**) TIMP-2 (**c**) TIMP-3 (**d**) TIMP-4. Data represents median upper and lower quartiles. Each dot represents BAL concentration of individual MMP in a specific patient, *n* = 24 for COPD and 8 for preserved lung function. * *p* < 0.05 using Mann-Whitney *U* test

We previously reported significantly raised concentrations of MMP-3, -8, -9 and -10 in COPD [15]. TIMP-1 and -2 were found in abundant concentrations within BAL (Fig. 2), while TIMP-4 was found in lower concentrations. TIMP-2 and -4 were significantly increased in COPD subjects while there was no significant difference between groups for TIMP-1 and TIMP-3.

### Emphysema sub-types and MMPs and TIMPs

We performed a systematic analysis of associations between emphysema sub-types and MMP and TIMP BAL concentrations in the whole cohort. MMP-3 and -10 had significant associations with all emphysema sub-types apart from mild CLE. MMP -7 and -8 had significant associations with all emphysema sub-types apart from mild CLE and PLE (Table 2). MMP-9 had significant associations with moderate CLE and PSE. MMP-12 did not exhibit any significant associations with emphysema sub-types. TIMP-4 had significant associations with moderate and severe CLE and PSE. No other TIMPs had associations with emphysema sub-types (Table 3).

**Table 2:** Spearman’s correlation analysis between MMPs and emphysema sub-types

	MMP-3	MMP-7	MMP-8	MMP-9	MMP-10	MMP-12
Mild CLE	−0.09	−0.11	−0.10	−0.8	0.05	−0.30
Moderate CLE	0.45*	0.43*	0.49*	0.42*	0.41*	0.15
Severe CLE	0.52**	0.39*	0.40*	0.33	0.38*	0.11
Panlobular	0.56**	0.34	0.33	0.29	0.43*	0.22
Paraseptal	0.50**	0.49**	0.46**	0.42*	0.44*	0.12
Non-emphysema	−0.45*	−0.39*	−0.41*	−0.36*	−0.45*	−0.44

Spearman’s rho values given

*N* = 31. *p < 0.05; **p < 0.01

**Table 3:** Spearman’s correlation analysis between TIMPs and emphysema sub-types

	TIMP-1	TIMP-2	TIMP-3	TIMP-4
Mild CLE	−0.04	−0.21	−0.23	−0.18
Moderate CLE	0.03	0.31	0.20	0.37*
Severe CLE	0.05	0.29	0.18	0.36*
Panlobular	−0.15	0.22	0.21	0.28
Paraseptal	−0.01	0.31	0.24	0.37*
Non-emphysema	−0.07	−0.31	−0.19	−0.36*

Spearman’s rho values given

*N* = 31. *p < 0.05

### MMPs/TIMPs ratios and emphysema sub-types

To understand the role of a proteinase/antiproteinase imbalance we investigated the MMP/TIMP ratios. Ratios were significantly increased in COPD subjects for MMP-8/TIMP-1, -2, -3, -4, MMP-9/TIMP-1 and MMP-10/TIMP-1 and -2 (Additional file 1).

We also investigated associations between emphysema sub-types and MMP/TIMP ratios (Tables 4, 5, 6 and 7). Mild CLE did not have any significant associations with MMP/TIMP ratios. Multiple MMP/TIMP ratios had associations with all the other tissue sub-types.

**Table 4:** Spearman’s correlation analysis between MMPs/TIMP1 ratios and emphysema sub-types

	MMP-3/TIMP-1	MMP-7/TIMP-1	MMP-8/TIMP-1	MMP-9/TIMP-1	MMP-10/TIMP-1	MMP-12/TIMP-1
Mild CLE	−0.08	−0.06	−0.05	−0.03	0.01	−0.14
Moderate CLE	0.39*	0.39*	0.44*	0.34	0.43*	0.12
Severe CLE	0.44*	0.36*	0.36*	0.28	0.40*	0.08
Panlobular	0.59***	0.43*	0.36*	0.31	0.55**	0.33
Paraseptal	0.47**	0.50**	0.42*	0.37*	0.47**	0.15
Normal	−0.37*	−0.35	−0.36*	−0.29	−0.44*	−0.04

Spearman’s rho values given

*N* = 31. *p < 0.05; **p < 0.01; ***p < 0.001

**Table 5:** Spearman’s correlation analysis between MMPs/TIMP2 ratios and emphysema sub-types

	MMP-3/TIMP-2	MMP-7/TIMP-2	MMP-8/TIMP-2	MMP-9/TIMP-2	MMP-10/TIMP-2	MMP-12/TIMP-2
Mild CLE	−0.07	−0.09	−0.06	−0.02	0.10	−0.01
Moderate CLE	0.35	0.22	0.42*	0.27	0.37*	−0.05
Severe CLE	0.44*	0.25	0.35	0.23	0.33	−0.10
Panlobular	0.56**	0.32	0.32	0.26	0.46**	0.08
Paraseptal	0.44*	0.34	0.40*	0.31	0.38*	−0.04
Normal	−0.35	−0.18	−0.34	−0.23	−0.40*	0.13

Spearman’s rho values given

*N* = 31. *p < 0.05; **p < 0.01

**Table 6:** Spearman’s correlation analysis between MMPs/TIMP3 ratios and emphysema sub-types

	MMP-3/TIMP-3	MMP-7/TIMP-3	MMP-8/TIMP-3	MMP-9/TIMP-3	MMP-10/TIMP-3	MMP-12/TIMP-3
Mild CLE	−0.04	0.02	−0.09	0.01	0.14	0.04
Moderate CLE	0.36*	0.27	0.44*	0.21	0.32	−0.05
Severe CLE	0.40*	0.22	0.32	0.12	0.23	−0.12
Panlobular	0.44*	0.16	0.30	0.13	0.24	−0.04
Paraseptal	0.32	0.27	0.39*	0.17	0.27	−0.13
Normal	−0.30	−0.20	−0.33	−0.13	−0.32	0.15

Spearman’s rho values given

*N* = 31. *p < 0.05

**Table 7:** Spearman’s correlation analysis between MMPs/TIMP4 ratios and emphysema sub-types

	MMP-3/TIMP-4	MMP-7/TIMP-4	MMP-8/TIMP-4	MMP-9/TIMP-4	MMP-10/TIMP-4	MMP-12/TIMP-4
Mild CLE	0.03	−0.02	−0.06	0.03	0.16	0.01
Moderate CLE	0.35	0.25	0.43*	0.15	0.30	−0.12
Severe CLE	0.41*	0.20	0.32	0.07	0.22	−0.21
Panlobular	0.54**	0.24	0.29	0.11	0.36*	0.01
Paraseptal	0.42*	0.32	0.38*	0.16	0.32	−0.14
Normal	−0.35	−0.20	−0.32	−0.09	−0.34	0.21

Spearman’s rho values given

*N* = 31. *p < 0.05; **p < 0.01

## Discussion

Using LHE patterns analysed on HRCT images, we successfully measured emphysema sub-types in mild/moderate COPD subjects and ex/current smokers with preserved lung function. The most prevalent tissue subtypes in COPD subjects were mild and moderate CLE and non-emphysematous tissue, whilst severe CLE, PSE and PLE were less frequently present. Furthermore, all emphysema sub-types, apart from mild CLE, had associations with multiple MMPs, particularly the stromelysins MMP-3 and MMP-10, implicating these proteases in the tissue destruction that occurs in these sub-types of emphysema. Interestingly, mild CLE was found in substantial quantities in subjects with and without airflow obstruction and exhibited different properties from the other sub-types of emphysema showing no associations with MMPs.

Emphysema is an important pathological feature of COPD, contributing directly to airflow obstruction and is associated with mortality and worse outcomes [25–27]. LHE CT analysis determines the distribution of the three main emphysema sub-types throughout the lungs and shows stronger associations with physiological and functional measures than current CT emphysema estimation (%LAA_<-950_) [2]. In our mild-moderate COPD subjects, the predominant emphysema sub-types were mild and moderate CLE, with only small quantities of severe CLE present, which is in keeping with previous work [2, 3]. CLE is an abnormal enlargement of air-spaces centred on the respiratory bronchiole and is the classical form associated with smoking [3, 5]. Only small amounts of PLE and PSE were present in COPD subjects which is consistent with other studies [2, 3, 6]. Panlobular emphysema is an abnormal enlargement of airspaces distributed throughout the pulmonary lobule and has been associated with A1ATD and more severe disease [3, 5]. It is therefore unsurprising that PLE was found in such low quantities in our cohort. Paraseptal emphysema refers to emphysematous change adjacent to a pleural surface and is the least well understood form of emphysema and shows no relationship with COPD symptoms [3] or smoking history [4].

The underlying mechanisms driving the evolution of emphysema sub-types are poorly understood. MMPs are proteolytic enzymes implicated in the destruction of the pulmonary extra-cellular matrix (ECM). We have previously reported that overall emphysema had associations with MMP-3, -7 and -10 and CT markers of small airways disease had associations with MMP-3, -7, -8, -9, -10 and -12 [15]. MMP-9 polymorphisms have been associated with CLE [10], but ours is the first study evaluating the contribution of MMPs to different emphysema sub-types in detail. MMP-3 and -10 had significant associations with all forms of emphysema (apart from mild CLE) while MMP-7, -8 and -9 had associations with multiple sub-types. MMPs are inhibited by four endogenous inhibitors, the TIMPs which bind with MMPs in a 1:1 manner [28]. TIMP-3 null mice develop emphysema [29] while human studies show TIMP-1 and -2 are raised in the airways of COPD subjects [13, 14, 19, 20] and TIMP-2 polymorphisms are associated with CLE [10]. We found significantly increased TIMP-2 and TIMP-4 in COPD subjects and also found that TIMP-4 had positive associations with several emphysema sub-types. This suggests that TIMPs are raised in COPD and emphysema although their activity and specificity are poorly understood. An imbalance between MMPs and TIMPs may be more important than absolute concentrations although previous studies have not demonstrated this [20, 23], or alternatively MMPs may be acting in the immediate pericellular environment and therefore not inhibited by TIMPs. In contrast to this previous data, we found a number of ratios were increased in COPD and a number had significant associations with emphysema sub-types.

Perhaps the most interesting results from this study were those relating to mild CLE. All subjects, irrespective of whether they had airflow obstruction, had evidence of mild CLE with median values above 30 %. This is consistent with other studies which have demonstrated emphysematous change in smokers without airflow obstruction [3, 4]. Using LHE CT analysis, Castaldi also described high rates of mild CLE in healthy smokers, linking it with degree of airflow obstruction and functional capacity [2]. This suggests that mild CLE is a genuine tissue abnormality and occurs prior to the development of airflow obstruction and a key question is whether this progresses into more significant disease. Unlike more severe forms of emphysema we found no associations between MMPs and mild CLE. When correcting for the other tissue sub-types, using partial correlation (data not shown) no associations between mild CLE and MMPs were found, suggesting mild CLE genuinely does not have any associations with MMPs. We propose a possible explanation for this may be that mild emphysema is ubiquitous in smokers and ex-smokers and occurs via a non-MMP derived mechanism, such as oxidative stress secondary to cigarette smoke exposure. It may be that only subjects with significant MMP activity develop more advanced emphysema and our results suggest MMP-3 and -10 are the most important in this process. These MMPs are stromelysins, mainly degrading collagen and proteoglycans, important constituents of the pulmonary ECM [22]. In addition MMP-7, -8 and -9 had associations with moderate CLE and PSE and MMP-7 and -8 also had associations with severe CLE. MMP-7 is an elastase, MMP-8 a collagenase and MMP-9 a gelatinase and together can degrade all components of the pulmonary ECM [22]. Given that individual MMPs have different substrate specificity, it may be proposed that particular MMPs are responsible for each sub-type of emphysema. However, our results do not support this as the profile of MMP expression is broadly similar across all emphysema sub-types, apart from mild CLE. Further mechanistic work is required to understand this is in more detail and longitudinal studies are needed to understand how mild CLE and the other emphysema sub-types progress and whether this can predicted by MMP concentrations.

The main limitation of this study was the small sample size and associated limited statistical power. Despite this we found strong evidence of associations between MMPs and emphysema sub-types. Another limitation is the multiple comparisons made in this study. Excluding Table 1, 70 out of 238 comparisons were significant, far more than the 12 associations expected to be significant by chance, suggesting genuine associations. Additionally, all significant results were in the same, expected direction for each MMP/TIMP and emphysema subtype comparison. Due to the need to perform bronchoscopy, our study consisted of patients with mild and moderate COPD, with only limited amounts of emphysema. It is unknown whether these results would be similar in a more severe cohort. Furthermore, the lack of a validation population limits the generalizability of the findings. Using LHE CT analysis we could only determine emphysema sub-types throughout the entire lungs rather than on a lobar basis and further work will involve refining this analysis to include lobar measurements. However we have previously shown that comparing BAL analysis to whole lung CT parameters is a valid technique [15]. Finally quantitative measures of MMPs and ratios with TIMPs do not necessarily equate to enzymatic activity. We are developing techniques to generate in-situ zymography data which will be the next step in understanding the aetiology of emphysema.

## Conclusion

In conclusion, mild and moderate CLE were the predominant forms of tissue sub-types measured in a cohort of mild/moderate COPD subjects. Multiple MMPs were associated with moderate and severe CLE, paraseptal and panlobular emphysema. MMP-3 and -10 had associations with all of these sub-types, suggesting they play an important part in the tissue destruction seen in these sub-types of emphysema. Mild CLE was common in both subjects with COPD and those with preserved lung function and unlike other tissue sub-types did not have any associations with MMPs. MMP activity may explain why some subjects progress to more severe forms of emphysema and increased understanding of these specific mechanisms may help inform our knowledge of disease progression and treatment options.

## Abbreviations

A1ATD, alpha-1-antitrypsin deficiency; BAL, bronchoalveolar lavage; CLE, centrilobular emphysema; COPD, chronic obstructive pulmonary disease; ECM, extra-cellular matrix; FEV1, Forced expiratory volume in 1 s; FVC, Forced vital capacity; LHE, local histogram-based emphysema patterns; MMP, matrix metalloproteinase; PLE, panlobular emphysema; PSE, paraseptal emphysema; TIMP, Tissue inhibitor of MMPs

## Additional file

Additional file 1: Sensitivity data for MMP and TIMP luminex assays. MMP/TIMP ratios in BAL in COPD and PLF subjects. (DOCX 171 kb)
