# Supplementary material for: Distinct emphysema subtypes defined by quantitative CT analysis are associated with specific pulmonary matrix metalloproteinases
Source: Respir Res. 2016 Jul 26;17:92. doi: 10.1186/s12931-016-0402-z (PMC4962504; doi:10.1186/s12931-016-0402-z)
Supplement: Additional file 1: — Sensitivity data for MMP and TIMP luminex assays. MMP/TIMP ratios in BAL in COPD and PLF subjects. (DOCX 171 kb) [file 12931_2016_402_MOESM1_ESM.docx]

Supplementary Data

**Supplementary methods**

The sensitivities for the MMP Luminex assays were MMP-1 1.1pg/ml, MMP-2 12.6pg/ml, MMP-3 7.3pg/ml, MMP-7 6.6pg/ml, MMP-8 16.6pg/ml, MMP-9 13.7pg/ml, MMP-10 3.2pg/ml, MMP-12 0.7pg/ml, MMP-13 63.5pg/ml, EMPRIN 5.6pg/ml.

The sensitivities for the TIMP Luminex assays were TIMP-1 3.43 pg/ml, TIMP-2 40.1pg/ml, TIMP-3, 20.0pg/ml, TIMP-4 0.28pg/ml.

**Supplementary Figure**

**Supplementary Figure** – MMP:TIMP ratios in BAL in COPD and preserved lung function. (A) MMP-3:TIMP-1 (B) MMP-3:TIMP-2 (C)MMP-3:TIMP-3 (D) MMP-3:TIMP-4 (E) MMP-7:TIMP-1 (F) MMP-7:TIMP-2 (G)MMP-7:TIMP-3 (H) MMP-3:TIMP-4 (I) MMP-8:TIMP-1 (J) MMP-8:TIMP-2 (K)MMP-8:TIMP-3 (L) MMP-8:TIMP-4 (M) MMP-9:TIMP-1 (N) MMP-9:TIMP-2 (O)MMP-9:TIMP-3 (P) MMP-9:TIMP-4 (Q) MMP-10:TIMP-1 (R) MMP-10:TIMP-2 (S)MMP-10:TIMP-3 (T) MMP-10:TIMP-4 (U) MMP-12:TIMP-1 (V) MMP-12:TIMP-2 (W)MMP-12:TIMP-3 (X) MMP-12:TIMP-4. Data represents median with IQ range. Each dot represents BAL ratio of MMP/TIMP in a specific patient, n=24 for COPD and 8 for preserved lung function. * p<0.05 ** p<0.01 ***p<0.001 using Mann-Whitney U test.
